# lncRNA DLEU1 Modulates Proliferation, Inflammation, and Extracellular Matrix Degradation of Chondrocytes through Regulating miR-671-5p

**DOI:** 10.1155/2022/1816217

**Published:** 2022-05-18

**Authors:** Xiangkun Wu, Shuai Yin, Lihua Yan, Yongxi Liu, Lilin Shang, Jun Liu

**Affiliations:** ^1^Department of Orthopaedic Surgery, Nanyang Second People's Hospital, Nanyang, Henan, China 473000; ^2^Graduate School of Tianjin Medical University, Tianjin, China; ^3^Department of Medical Oncology, Nanyang Second People's Hospital, Nanyang, Henan, China 473000; ^4^Department of Joint Surgery, Tianjin Hospital, Tianjin 300211, China

## Abstract

Long noncoding RNAs (lncRNAs) have been shown to be involved in the development of osteoarthritis. However, the expression, function, and mechanism of DLEU1 in OA development remain largely unclear. The present reference demonstrates that DLEU1 is overexpressed in OA specimens compared to control cartilages. Inflammatory cytokines IL-1*β*, TNF-*α*, and IL-6 induce DLEU1 expression in chondrocytes. Ectopic expression of DLEU1 induces chondrocyte proliferation, degradation of ECM, and inflammation mediators such as IL-6, IL-8, and TNF-*α* secretion. Moreover, we demonstrated that DLEU1 targets miR-671-5p expression in chondrocytes. Overexpression of DLEU1 suppresses miR-671-5p expression in chondrocytes. The expression of miR-671-5p is decreased in OA specimens compared to control cartilages. There is a negative correlation between the expression of miR-671-5p and DLEU1 in OA specimens. Inflammatory mediators IL-1*β*, TNF-*α*, and IL-6 suppress miR-671-5p expression in OA specimens. Elevated expression of miR-671-5p suppresses chondrocyte proliferation, degradation of ECM, and secretion of inflammation mediators. DLEU1 overexpression promotes chondrocytes proliferation, degradation of ECM, and secretion of inflammation mediators via regulating miR-671-5p. These results suggested that DLEU1 acts as one destructive role in OA development via regulating miR-671-5p.

## 1. Introduction

Osteoarthritis is one degenerative joint disease characterized by deterioration in cartilage, synovial inflammation, chondrocyte hypertrophy, and subchondral sclerosis [1–6]. Osteoarthritis is one leading account of disability, pain, and working life shortening [7, 8]. Multiple causes such as strain, inflammation, aging, trauma, obesity, and congenital malformation contribute to progression of OA [9–12]. However, no definite treatment is available that could alter its development.

Long noncoding RNAs (lncRNAs) are one category of noncoding RNAs that are longer than 200 nucleotides, which act as a sponge for miRNA and suppress miRNA expression [13–17]. Growing references have showed that aberrant lncRNA expression is correlated with several diseases including cancer, myocardial ischemia, immunity- and inflammation-related disease, intervertebral disc degeneration, and OA [18–22]. For example, Jiang et al. [23] demonstrated that SNHG5 induced chondrocyte proliferation and suppressed apoptosis in osteoarthritis through modulating the H3F3B/miR-10a-5p axis. Zhou et al. [24] found that lncRNA PCAT-1 modulated chondrocyte apoptosis through sponging miR-27b-3p in osteoarthritis. lncRNAs are involved in cell functions such as cell apoptosis, differentiation, proliferation, metabolism, and invasion [25–28]. Recently, a new lncRNA DLEU1 has been confirmed to act crucial roles in pathologic development of renal cell carcinoma, osteosarcoma, glioma, bladder cancer, and cervical cancer [29–33]. However, the expression, function, and mechanism of DLEU1 in OA development remain largely unclear. Recently, Chen et al. showed that circ-PTTG1IP promotes migration, proliferation, inflammatory response, and invasion of fibroblast-like synoviocytes in the rheumatoid arthritis [34]. Furthermore, Ma et al. also found that paeoniflorin inhibited rheumatoid arthritis progression through regulating the circ-FAM120A/MDM4/miR-671-5p axis [35]. Moreover, DLEU1 regulated osteosarcoma cell migration, invasion, and migration through targeting miR-671-5p [31].

We studied the role of DLEU1 in OA. Firstly, we indicated that DLEU1 is overexpressed in OA specimens compared to control cartilages. Inflammatory cytokines IL-1*β*, TNF-*α*, and IL-6 induce DLEU1 expression in chondrocytes. Ectopic expression of DLEU1 induces chondrocyte proliferation, degradation of ECM, and secretion of inflammation mediators, such as IL-6, IL-8, and TNF-*α*.

## 2. Materials and Methods

### 2.1. Tissue Specimens

OA cartilages were collected from OA cases that underwent TKA (total knee arthroplasty), and control cartilages were obtained from cases due to the fracture of knee joint without rheumatoid arthritis or OA. Each case was given an informed consent, and our study was agreed by the Clinical Ethics Committee of our hospital. The demographic and clinical characteristics of OA patients are indicated in Table 1.

### 2.2. Chondrocyte Culture and Transfection

Human chondrocytes were obtained from Shanghai Chinese Academy of Sciences, and these cells were cultured in the RPMI-1640 medium containing antibiotics and fetal bovine serum (FBS). pcDNA-DLEU1 and control and miR-671-5p mimic and scramble mimic were collected from GenePharma. Cell transfection was carried out with using Lipofectamine 2000 (Invitrogen, USA) following the protocol.

### 2.3. Luciferase Reporter Assay

Fragment of DLEU1 3′-UTR was cloned into downstream of pMIR-REPORT plasmid, named wild-type (WT) DLEU1. To build mutant DLEU1 3′-UTR reporter, seed region of DLEU1 3′-UTR was mutated, named mutated (mut) DLEU1. Cells were cotransfected with miR-671-5p mimic or scramble and mutant DLEU1 3′-UTR or mut-DLEU1 using Lipofectamine 2000 (Invitrogen, USA). Luciferase activity was carried out with luciferase reporter analysis (Promega).

### 2.4. Cell Viability Assay

Cell viability was detected using CCK-8 reagent (Dojindo, Japan) following the typical protocol. Cells were cultured in the 96-well dish and 10 *μ*l CCK-8 fluids were added into each well. After incubation for additional 3 hours, the absorbance was read with a microplate reader at 450 nm.

### 2.5. RT-qPCR

Total RNA from chondrocytes or cartilage samples was separated by Trizol kit (Invitrogen, USA) according to the protocol. Real-time qPCR was utilized to determine the expression of miR-671-5p, DLEU1, aggrecan, collagen II, ADAMTS-5, and MMP-3 using SYBRTM Green kit (Applied Biosystems, CA) on ABI7500 PCR System (Applied Biosystems, USA). U6 and GAPDH were performed for internal controls. Results were calculated by utilizing the 2^−△△CT^ way. Primers were indicated as follows: U6 primers (forward, 5′-CTCGCT TCG GCA GCA CA-3′and reverse, 5′-AACGCT TCA CGA ATT TGCGT-3′), DLEU1 (forward 5′-CCAGC CCACA GGCAT TTAGT-3′and reverse, 5′-GTTCC GAGG CTTAA GTGCGA-3′), and GAPDH (forward, 5′-AAGTGGTCGTTGAGGGCAATG-3′and reverse, 5′-CTGGGCTACACTGAGCACC-3′).

### 2.6. ELISA

The culture supernatant was obtained after chondrocytes were treated. The IL-6, IL-8, and TNF-*α* concentration in the supernatant was detected with ELISA reagent (R&D Systems, UK) following the standard protocol.

### 2.7. Statistical Analysis

SPSS 18.0 software (Chicago, IL) was processed for statistical assay. Results were indicated as means ± SD. Student's *t*-test was applied to detect statistical difference between two groups. Statistically significance was set to *p* < 0.05.

## 3. Results

### 3.1. DLEU1 Is Overexpressed, and miR-671-5p Is Decreased in OA Specimens

**Figure 1:**
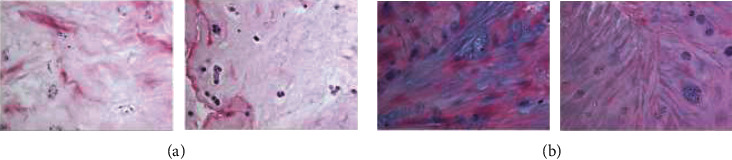
The pathology of control cartilages and OA cartilages is shown in (a) and (b).

**Figure 2:**
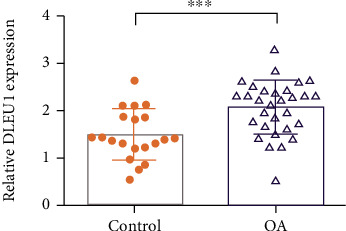
DLEU1 was overexpressed in OA specimens. The DLEU1 expression in the control cartilages and OA cartilages was detected by qRT-PCR assay. GAPDH was used as the internal control. ^∗∗∗^*p* < 0.001.

**Figure 3:**
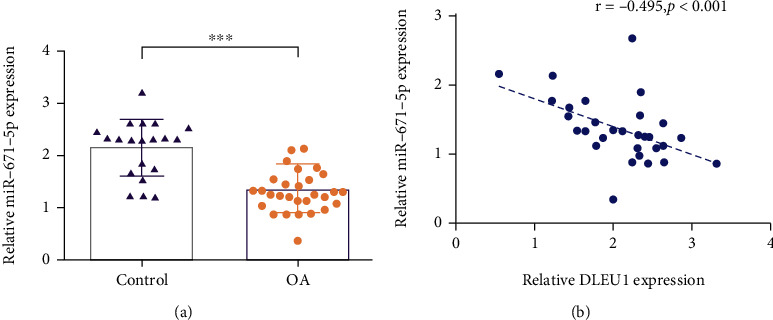
miR-671-5p was decreased in OA specimens. (a) The miR-671-5p expression in the control cartilages and OA cartilages was detected by qRT-PCR assay. (b) There was a negative correlation expression between miR-671-5p and DLEU1 in OA specimens. ^∗∗∗^*p* < 0.001.

The pathology of control cartilages and OA cartilages is shown in Figures 1(a) and 1(b). qRT-PCR assay was carried out to examine DLEU1 expression in control cartilages and OA cartilages. As indicated in Figure 2, DLEU1 is overexpressed in OA specimens compared to control cartilages. Moreover, qRT-PCR assay was carried out to examine miR-671-5p expression in control cartilages and OA cartilages. As indicated in Figure 3(a), miR-671-5p is decreased in OA specimens compared to control cartilages. There is a negative correlation between expression of miR-671-5p and DLEU1 in OA specimens (Figure 3(b)).

### 3.2. Inflammatory Cytokines IL-1*β*, TNF-*α*, and IL-6 Induce DLEU1 and Suppress miR-671-5p Expression

**Figure 4:**
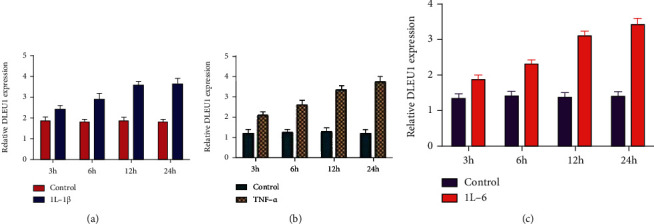
Inflammatory cytokines IL-1*β*, TNF-*α*, and IL-6 induced DLEU1 expression. (a) Inflammatory cytokine IL-1*β* increased DLEU1 expression in chondrocytes. (b) The expression of DLEU1 was measured by qRT-PCR analysis. (c) IL-6 induced DLEU1 expression in chondrocytes.

**Figure 5:**
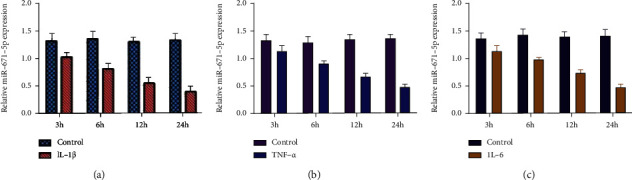
Inflammatory mediators IL-1*β*, TNF-*α*, and IL-6 suppressed miR-671-5p expression. (a) The expression of miR-671-5p was detected by qRT-PCR assay. (b) TNF-*α* suppressed miR-671-5p expression in chondrocytes. (c) IL-6 inhibited miR-671-5p expression in chondrocytes.

We found that inflammatory cytokines IL-1*β*, TNF-*α*, and IL-6 increase DLEU1 expression in chondrocytes (Figures 4(a)–4(c)). We indicated that inflammatory cytokines IL-1*β*, TNF-*α*, and IL-6 decrease miR-671-5p expression in chondrocytes (Figures 5(a)–5(c)).

### 3.3. Ectopic Expression of DLEU1 Induces Chondrocyte Proliferation, Degradation of ECM, and Secretion of Inflammation Mediators

**Figure 6:**
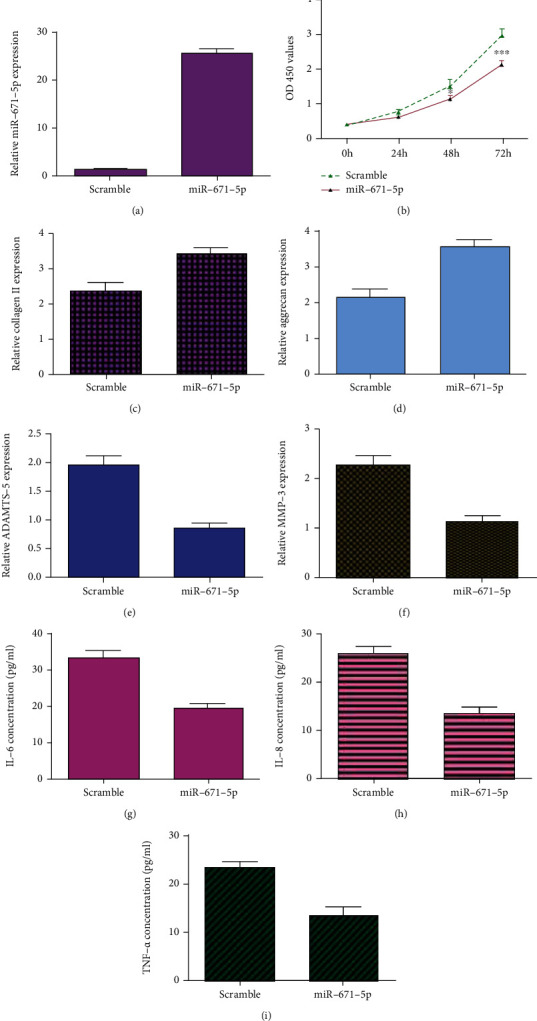
Ectopic expression of DLEU1 induced chondrocyte viability, degradation of ECM, and inflammation mediator secretion. (a) The expression of DLEU1 was measured by qRT-PCR analysis. (b) Ectopic expression of DLEU1 promoted cell viability in the chondrocytes using CCK-8 assay. (c) Elevated expression of DLEU1 suppressed collagen II expression in the chondrocytes. (d) The expression of aggrecan was detected by qRT-PCR assay. (e) Overexpression of DLEU1 induced ADAMTS-5 expression in the chondrocytes. (f) The expression of MMP-3 was measured by qRT-PCR assay. (g) DLEU1 overexpression promoted IL-6 expression in the chondrocytes using ELISA. (F) The expression of IL-8 was measured by ELISA. (i) The expression of TNF-*α* was measured by ELISA. ^∗^*p* < 0.05, ^∗∗^*p* < 0.01, and ^∗∗∗^*p* < 0.001.

The expression of DLEU1 is upregulated in chondrocytes transfected with pcDNA-DLEU1 plasmid (Figure 6(a)). Ectopic expression of DLEU1 promotes cell proliferation in the chondrocytes using CCK-8 assay (Figure 6(b)). Elevated DLEU1 expression suppresses the expression of collagen II (Figure 6(c)) and aggrecan (Figure 6(d)) in chondrocytes. Overexpression of DLEU1 induces ADAMTS-5 (Figure 6(e)) and MMP-3 (Figure 6(f)) expression in chondrocytes. Furthermore, DLEU1 overexpression promotes IL-6 (Figure 6(g)), IL-8 (Figure 6(h)), and TNF-*α* (Figure 6(i)) expression in chondrocytes using ELISA.

### 3.4. Elevated Expression of miR-671-5p Suppresses Chondrocyte Viability, Degradation of ECM, and Secretion of Inflammation Mediators

**Figure 7:**
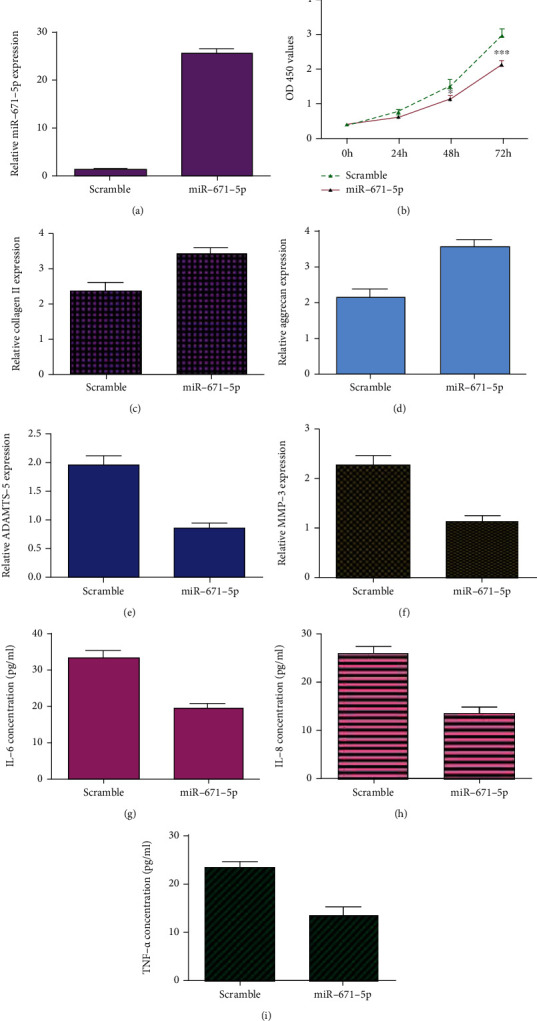
Elevated expression of miR-671-5p suppressed chondrocyte viability, degradation of ECM, and inflammation mediator secretion. (a) The expression of miR-671-5p was measured by qRT-PCR analysis. (b) Ectopic expression of miR-671-5p inhibited cell viability in the chondrocytes using CCK-8 assay. (c) Elevated expression of miR-671-5p enhanced collagen II expression in the chondrocytes. (d) The expression of aggrecan was detected by qRT-PCR assay. (e) Overexpression of miR-671-5p suppressed ADAMTS-5 expression in the chondrocytes. (f) The expression of MMP-3 was measured by qRT-PCR assay. (g) miR-671-5p overexpression inhibited IL-6 expression in the chondrocytes using ELISA. (h) The expression of IL-8 was measured by ELISA. (i) The expression of TNF-*α* was measured by ELISA. ^∗^*p* < 0.05 and ^∗∗∗^*p* < 0.001.

The expression of miR-671-5p is upregulated in chondrocytes transfected with miR-671-5p mimic (Figure 7(a)). Ectopic expression of miR-671-5p inhibits cell viability in the chondrocytes using CCK-8 assay (Figure 7(b)). Elevated miR-671-5p expression enhances expression of collagen II (Figure 7(c)) and aggrecan (Figure 7(d)) in the chondrocytes. Overexpression of miR-671-5p suppresses ADAMTS-5 (Figure 7(e)) and MMP-3 (Figure 7(f)) expression in the chondrocytes. Furthermore, miR-671-5p overexpression inhibits IL-6 (Figure 7(g)), IL-8 (Figure 7(h)), and TNF-*α* (Figure 7(i)) expression in the chondrocytes using ELISA.

### 3.5. DLEU1 Targets miR-671-5p Expression in Chondrocytes

**Figure 8:**
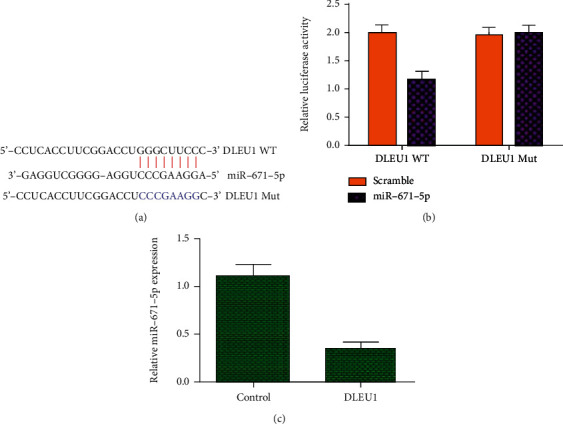
DLEU1 targeted miR-671-5p expression in chondrocytes. (a) Following the software (http://starbase.sysu.edu.cn/index.php), miR-671-5p may be a potential combining target gene of the DLEU1. (b) Luciferase reporter analysis was carried out to study the relationship between miR-671-5p and DLEU1. The data indicated that cotransfection with miR-671-5p mimic and DLEU1-WT decreased luciferase activities when compared to miR-671-5p mimic and DLEU1-mut. (c) Overexpression of DLEU1 suppressed miR-671-5p expression in chondrocytes. ^∗∗^*p* < 0.01.

Following the software (http://starbase.sysu.edu.cn/index.php), miR-671-5p may be a potential combining target gene of the DLEU1 (Figure 8(a)). Luciferase reporter analysis was carried out to study the relationship between miR-671-5p and DLEU1. The data indicated that cotransfection with miR-671-5p mimic and DLEU1-WT decreases luciferase activities when compared to miR-671-5p mimic and DLEU1-mut (Figure 8(b)). In addition, qRT-PCR assay data showed that overexpression of DLEU1 suppresses miR-671-5p expression in chondrocytes (Figure 8(c)).

### 3.6. DLEU1 Overexpression Promotes Chondrocyte Viability, Degradation of ECM, and Secretion of Inflammation Mediators via Regulating miR-671-5p

**Figure 9:**
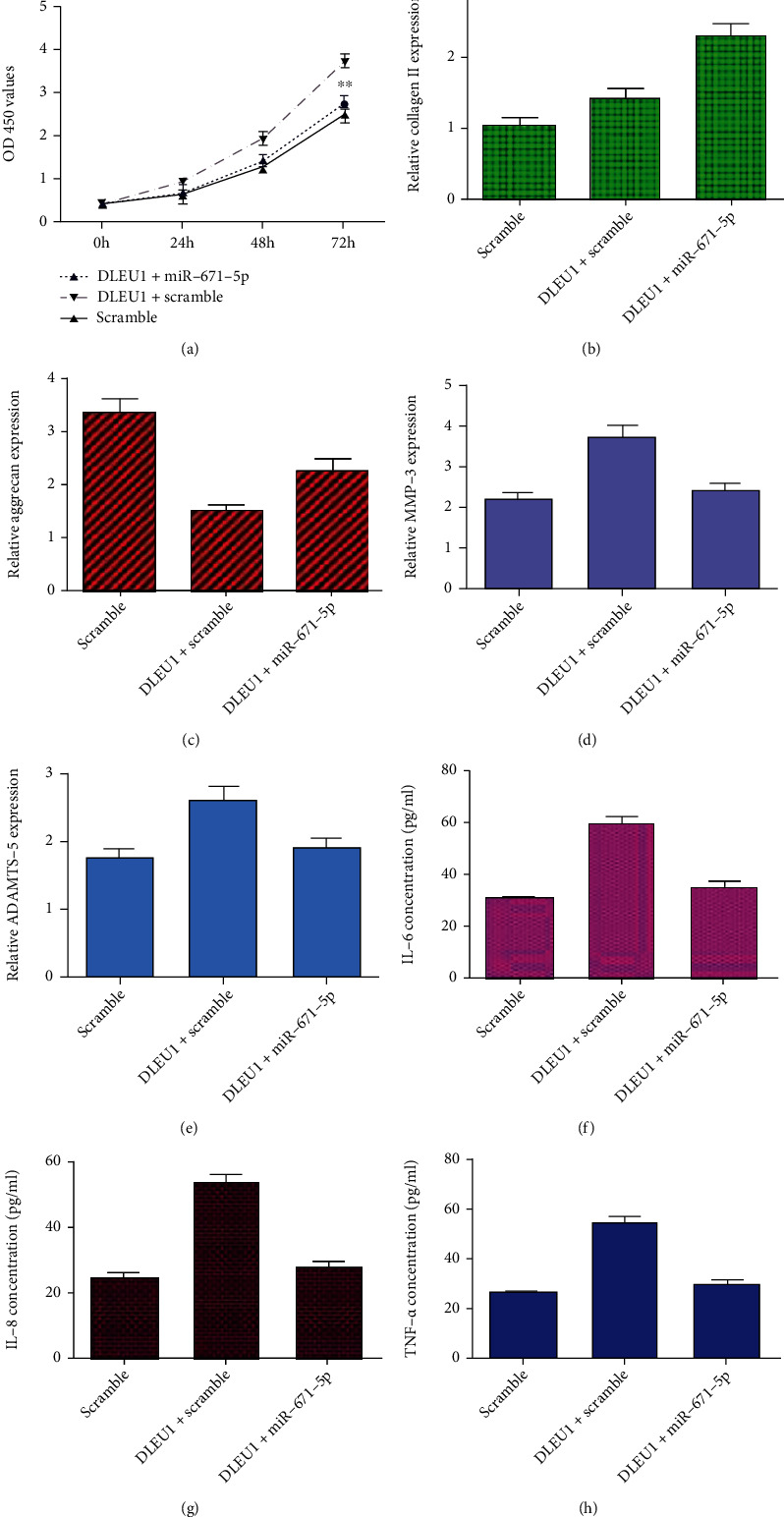
DLEU1 overexpression promoted chondrocyte proliferation, degradation of ECM, and inflammation mediator secretion via regulating miR-671-5p. (a) The cell proliferation was analyzed by CCK-8 assay. (b) The expression of collagen II was detected by qRT-PCR analysis. (c) The expression of aggrecan was detected by qRT-PCR analysis. (d) The expression of ADAMTS-5 was detected by qRT-PCR analysis. (e) The expression of MMP-3 was detected by qRT-PCR analysis. (f) The expression of IL-6 was measured by ELISA. (g) The expression of IL-8 was measured by ELISA. (h) The expression of TNF-*α* was measured by ELISA. ^∗^*p* < 0.05 and ^∗∗^*p* < 0.01.

To learn whether DLEU1 overexpression promoted chondrocyte viability, degradation of ECM, and secretion of inflammation mediators via regulating miR-671-5p, we performed the rescued experiments. We indicated that miR-671-5p overexpression suppresses cell growth in the DLEU1-overexpressing chondrocytes (Figure 9(a)). Elevated expression of miR-671-5p enhances collagen II (Figure 9(b)) and aggrecan (Figure 9(c)) expression in the DLEU1-overexpressing chondrocytes. Overexpression of miR-671-5p suppresses expression of ADAMTS-5 (Figure 9(d)) and MMP-3 (Figure 9(e)) in the DLEU1-overexpressing chondrocytes. Furthermore, miR-671-5p overexpression inhibits expression of IL-6 (Figure 9(f)), IL-8 (Figure 9(g)), and TNF-*α* (Figure 9(h)) in the DLEU1-overexpressing chondrocytes using ELISA.

## 4. Discussion

Emerging reports have supported that lncRNAs are aberrantly expressed in many physiological and pathological procedures, including OA. In this research, we studied the role of DLEU1 in OA. Firstly, we indicated that DLEU1 is overexpressed in OA specimens compared to control cartilages. Inflammatory cytokines IL-1*β*, TNF-*α*, and IL-6 induce DLEU1 expression in chondrocytes. Ectopic expression of DLEU1 induces chondrocyte proliferation, degradation of ECM, and secretion of inflammation mediators such as IL-6, IL-8, and TNF-*α*. Moreover, we demonstrated that DLEU1 targets miR-671-5p in chondrocytes. Overexpression of DLEU1 suppresses miR-671-5p expression in chondrocytes. The expression of miR-671-5p is decreased in OA specimens compared to control cartilages. There is a negative correlation between expressions of miR-671-5p and DLEU1 in OA specimens. Inflammatory mediators IL-1*β*, TNF-*α*, and IL-6 suppresses miR-671-5p expression in OA specimens. Elevated expression of miR-671-5p suppresses chondrocyte proliferation, degradation of ECM, and secretion of inflammation mediators. DLEU1 overexpression promotes chondrocyte proliferation, degradation of ECM, and secretion of inflammation mediators via regulating miR-671-5p. These results suggested that DLEU1 acts one destructive role in OA development via regulating miR-671-5p.

Recently, studies have revealed that DLEU1 plays critical roles in the pathologic progression of renal cell carcinoma, glioma, osteosarcoma, cervical cancer, and bladder cancer [29–33]. For instance, DLEU1 was upregulated in glioblastoma and downregulated expression of DLEU1 suppressed glioblastoma cell growth and enhanced cell apoptosis partly via regulating miR-4429 [36]. Yue et al. [29] found that knockdown of DLEU1 suppressed renal cell carcinoma cell growth, invasion, and migration and impaired epithelial mesenchymal transition progression partly through modulating Akt. Chen et al. [31] demonstrated that DLEU1 was upregulated in osteosarcoma cells and specimens. DLEU1 downregulation decreased osteosarcoma cell migration, invasion, and migration. Moreover, DLEU1 was shown to be overexpressed in bladder cancer specimens and overexpression of DLEU1 promoted cell invasion and growth and cisplatin resistance via modulating miR-99b expression [32]. However, the expression, function, and mechanism of DLEU1 in OA development remain largely unclear. In the present reference, we firstly detected the expression of DLEU1 in OA specimens. Our results showed that DLEU1 is overexpressed in OA specimens compared to control cartilages. Inflammatory cytokines IL-1*β*, TNF-*α*, and IL-6 induce DLEU1 expression in chondrocytes. Ectopic expression of DLEU1 induces chondrocytes proliferation, degradation of ECM, and inflammation mediators such as IL-6, IL-8, and TNF-*α* secretion.

Accumulating studies have proved that lncRNA performed as ceRNA to modulate disease development via sponging miRNA [37, 38]. Wang and workmates showed that NEAT1 promoted chondrocyte inflammation and proliferation through regulating miR-181a [39]. Zhu and Jiang [40] showed that PART1 regulated cell apoptosis, proliferation, and degradation of extracellular matrix through modulating miR-373-3p expression. Chen et al. [41] demonstrated that MEG3 alleviated extracellular matrix degradation in chondrocytes via targeting miR-93. Furthermore, DLEU1 regulated osteosarcoma cell migration, invasion, and migration through targeting miR-671-5p [31]. Following the software (http://starbase.sysu.edu.cn/index.php), miR-671-5p may be a potential combining target gene of DLEU1. Luciferase reporter analysis was carried out to study the relationship between miR-671-5p and DLEU1. The data indicated that cotransfection with miR-671-5p mimic and DLEU1-WT decreases luciferase activities when compared to miR-671-5p mimic and DLEU1-mut (Figure 7(b)). In addition, qRT-PCR assay data showed that overexpression of DLEU1 suppresses miR-671-5p expression in chondrocytes. In addition, we showed that the expression of miR-671-5p is decreased in OA specimens compared to control cartilages. There is a negative correlation between expression of miR-671-5p and DLEU1 in OA specimens. Inflammatory mediators IL-1*β*, TNF-*α*, and IL-6 suppress miR-671-5p expression in OA specimens. Elevated expression of miR-671-5p suppresses chondrocyte proliferation, degradation of ECM, and inflammation mediator secretion. DLEU1 overexpression promotes chondrocyte proliferation, degradation of ECM, and secretion of inflammation mediators via regulating miR-671-5p.

Our results defined that DLEU1 is overexpressed in OA specimens compared to control cartilages. DLEU1 overexpression promotes chondrocyte proliferation, degradation of ECM, and secretion of inflammation mediators via regulating miR-671-5p. These data suggested that DLEU1 acts a destructive role in OA development via regulating miR-671-5p expression.

## Figures and Tables

**Table 1:** Clinical and demographic characteristics of the study OA and control population.

Clinical data	OA	Control
Age (years)	67.6 ± 4.42	43.2 ± 5.24
Male/female (*n*)	19/11	13/7
Disease duration (years)	7.93 ± 3.35	0.82 ± 0.21
HSS score	49.87 ± 4.86	53.34 ± 4.35
CRP (mg/dl)	0.46 ± 0.16	0.31 ± 0.11
ESR (mm/h)	10.43 ± 4.56	11.35 ± 3.32

## Data Availability

No data were used to support this study.
